# Proof-of-concept model for instantaneous heart rate-drift correction during low and high exercise exertion

**DOI:** 10.3389/fphys.2024.1358785

**Published:** 2024-04-22

**Authors:** Gabriele B. Papini, Alberto G. Bonomi, Francesco Sartor

**Affiliations:** ^1^ Hospital Patient Monitoring, Royal Philips Electronics, Eindhoven, Netherlands; ^2^ Department of Electrical Engineering, Technical University Eindhoven, Eindhoven, Netherlands; ^3^ Clinical Affairs Office, Philips Medical Systems, Eindhoven, Netherlands; ^4^ Institute for Applied Human Physiology, Bangor University, Bangor, United Kingdom

**Keywords:** cardiovascular drift, training impulse, VO_2_ max, hysteresis, wearables

## Abstract

**Introduction:** This study aimed to model below and above anaerobic threshold exercise-induced heart rate (HR) drift, so that the corrected HR could better represent ${\dot{V}}_{O_{2}}$ kinetics during and after the exercise itself.

**Methods:** Fifteen healthy subjects (age: 28 ± 5 years; ${\dot{V}}_{O_{2}Max}$ : 50 ± 8 mL/kg/min; 5 females) underwent a maximal and a 30-min submaximal (80% of the anaerobic threshold) running exercises. A five-stage computational (i.e., delay block, new training impulse-calculation block, Sigmoid correction block, increase block, and decrease block) model was built to account for instantaneous HR, fitness, and age and to onset, increase, and decrease according to the exercise intensity and duration.

**Results:** The area under the curve (AUC) of the hysteresis function, which described the differences in the maximal and submaximal exercise-induced ${\dot{V}}_{O_{2}}$ and HR kinetics, was significantly reduced for both maximal (26%) and submaximal (77%) exercises and consequent recoveries.

**Discussion:** In conclusion, this model allowed HR drift instantaneous correction, which could be exploited in the future for more accurate ${\dot{V}}_{O_{2}}$ estimations.

## 1 Introduction

After a few minutes (e.g., 5–10 min) (Coyle, 1998) of submaximal aerobic or below anaerobic threshold exercise (BTE, e.g., 70% ${\dot{V}}_{O_{2}Max}$ ) at a constant workload (Hamilton et al., 1991), a progressive increase in the heart rate (HR) is observed. Invasive studies showed that this progressive increase in the HR is associated with a decrease in the stroke volume (SV) (Ekelund, 1967; Rowell, 1974). This phenomenon, where, for a given oxygen removal $\left(C_{aO_{2}}-C_{\bar{v}O_{2}}\right)$ , cardiac output (*CO* = *HR* ⋅ *SV*) matches ${\dot{V}}_{O_{2}}$ by an increase in the HR while SV decreases, follows the Fick equation (Eq. 1):

$${\dot{\;V}}_{O_{2}}=\left(↑HR\cdot ↓SV\right)\left(C_{aO_{2}}-C_{\bar{v}O_{2}}\right),$$ (1)

and it is referred to as cardiovascular (CV) drift. Hamilton et al. (1991) showed that during 2 h of cycling exercise at 70% ${\dot{V}}_{O_{2}Max}$ , the HR was elevated for at least two reasons: a response to the hypovolemic-induced decrease in SV to maintain adequate CO and a response to the increase in catecholamine circulating levels. In this respect, the continuous increase in catecholamine concentrations with exercise duration at a fixed ${\dot{V}}_{O_{2}}$ is well documented (Zouhal et al., 2008). Although ${\dot{V}}_{O_{2}}$ should match the exercise-induced energy requirements as determined by the exercise workload, a small ${\dot{V}}_{O_{2}}$ drift has been observed in prolonged aerobic submaximal exercises, and it is most probably related to increased liver gluconeogenesis (Hamilton et al., 1991). However, HR drift and ${\dot{V}}_{O_{2}}$ drift are notably different as they differ in origin, magnitude, onset, and kinetics. The first is mainly hemodynamically driven, while the second is mainly metabolically driven. Moreover, ${\dot{V}}_{O_{2}}$ drift (≈200 mL O_2_ in exercise ≥60 min) should not be confused with the slow component of ${\dot{V}}_{O_{2}}$ kinetics (1,000–1,500 mL ⋅ min^−1^), which arises when the submaximal exercise is above the anaerobic threshold, and it is mainly due to loss in muscle efficiency (Jones et al., 2011). Still, HR drift in those submaximal, yet above-anaerobic threshold, exercises shows a “slow component” that is similar to that observed for ${\dot{V}}_{O_{2}}$ , despite their different mediating factors (Pettitt et al., 2008; Zuccarelli et al., 2018). The ${\dot{V}}_{O_{2}}$ slow component is independent of the catecholamine levels, whereas the HR drift during above-threshold exercises is governed by neurohumoral events to match metabolic demands (Pettitt et al., 2008). Furthermore, heavy exercise-induced hyperthermia is another known cause of HR drift (Matsui et al., 1978).

**FIGURE 1:**
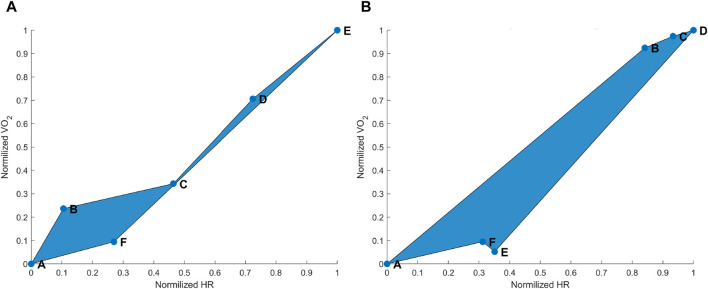
**(A)** Hysteresis derived from the maximal aerobic exercise (MAX) for a subject representative of the group. Data points: A = pre-MAX resting; B = pre-MAX self-selected speed (SSS) walking; C = beginning of MAX; D = half of MAX; E = end of MAX; and F = post-MAX resting. **(B)** Hysteresis derived from below anaerobic-threshold exercise (BTE) for a subject representative of the group. Data points: A = pre-BTE SSS walking; B = first phase of BTE (8th and 10th minute); C = middle phase of BTE (20th minute); D = end phase of BTE; E = post-BTE SSS walking; and F = post-BTE SSS + 1-km/h 2nd walking. The blue area represents the hysteresis when normalized VO_2_ is related to normalized heart rate (HR) values.

Interestingly, CV elevation persists during the post-exercise recovery phase, with SV and HR remaining depressed and elevated, respectively, for several minutes even after exercise termination [see Figure 7 in the study by Miyamoto et al. (1982)]. When studying the excess post-exercise oxygen consumption, it is clear that, after a consistent rapid recovery component, ${\dot{V}}_{O_{2}}$ stays elevated for several minutes proportionally to the exercise intensity and exercise duration (Borsheim and Bahr, 2003). The prolonged ${\dot{V}}_{O_{2}}$ recovery component accounts mainly for metabolic substrate replenishment, while the catecholamine levels may affect it indirectly (Borsheim and Bahr, 2003). Similar to what is argued for the exercise phase, ${\dot{V}}_{O_{2}}$ and HR do not exactly follow the same kinetics during recovery. Since the high level of post-exercise catecholamines is mainly due to higher exercise-induced secretion (Zouhal et al., 2008), it is also reasonable to think that HR “drift” during and after maximal aerobic exercise (MAX) is heavily influenced by catecholamines. Next to a high level of circulating catecholamines, the HR “drift,” or HR elevation, during the post-exercise recovery phase is affected by heat accumulation (Rowell et al., 1996) when facing dehydration (Astrand et al., 2003). In order to clearly show the discrepancy that we have so far introduced between HR and ${\dot{V}}_{O_{2}}$ drifts during and after maximal and submaximal exercise, these two variables can simply be plotted against each other. When ${\dot{V}}_{O_{2}}$ and HR values during exercise and recovery of both above or below anaerobic-threshold exercises are related to each other, a hysteresis trend can be observed (Figure 1). The existence of this hysteresis clearly shows the discrepancy between ${\dot{V}}_{O_{2}}$ and HR kinetics.

As we have previously shown, the relationship between ${\dot{V}}_{O_{2}}$ and HR does not have a purely academic interest, but it can be exploited for several applications, such as energy expenditure estimation (Altini et al., 2014; Bonomi et al., 2015; Kraal et al., 2016) and cardio–respiratory fitness assessment (Altini et al., 2016; Sartor et al., 2016; Bonomi et al., 2020). However, in light of the hysteresis formed by the kinetics of those two parameters, ${\dot{V}}_{O_{2}}$ cannot merely be estimated from the HR, and a correction should be applied. The correction should account for exercise intensity and duration and individual differences in body characteristics and aerobic fitness. Such models are not new in the literature; for instance, Banister in Calvert et al. (1976) introduced a model of cardiovascular endurance performance (Eq. 2), which is as follows:

$$\omega \left(t\right)=D\left(\Delta HR_{\mathrm{ratio}}\right)Y,$$ (2)

where *ω*(*t*) is the amount of training per session and is referred to as the training impulse (TRIMP), *D* is the exercise duration, Δ*HR*_*ratio*_ provided the personalized exercise intensity as it was calculated by dividing the difference between exercise HR and rest HR by HR reserve, and $Y=e^{b\left(\Delta HR_{\mathrm{ratio}}\right)}$ is a weighting factor that gave more weight to high-intensity training, where *b* reflects the exponential increase in blood lactate levels, as explained by Morton et al. (1990). This model is useful for predicting the cardiovascular effects, “fatigue,” and “fitness” of cyclical exercises, such as running, cycling, and rowing. Nevertheless, these effects refer to a complete training session, and the TRIMP model is affected by the HR drift. According to what we have introduced so far, it is easy to demonstrate how the same exercise stimulus (e.g., 10 km of running at a constant speed) performed by the same person at a given fitness level would lead to two very different TRIMPs when performed under either thermal comfort–euhydration conditions or under heat and dehydration. Moreover, as just mentioned, the TRIMP model provides a computation per training session, whereas the HR drift correction would require being activated and deactivated at the right time and instantaneously.

The aim of this study was to model low (below anaerobic threshold)- and high (above anaerobic threshold)-intensity components of exercise-induced HR drift so that the corrected HR could better represent ${\dot{V}}_{O_{2}}$ kinetics during and after the exercise itself, reducing, for instance, the area under the curve (AUC) of the hysteresis function between ${\dot{V}}_{O_{2}}$ and the HR.

## 2 Methods

### 2.1 Participants and study design

In order to develop a model to estimate ${\dot{V}}_{O_{2}}$ from the HR accounting for HR “drift” in exercises above and below the anaerobic threshold, 15 healthy adult subjects were recruited (Table 1) and asked to perform a graded maximal running test and a 30-min submaximal running test at 80% of their anaerobic threshold. The study protocol was approved by the Internal Ethics Committee of Philips Research, Eindhoven, in accordance with the Declaration of Helsinki.

**TABLE 1:** Participant characteristics.

Males = 15; females = 5	Mean ± SD
Age (years)	27.9 ± 5.4
Height (m)	1.79 ± 0.09
Weight (kg)	72.4 ± 10.2
VO_2_ max (mL/kg/min)	49.75 ± 8.40
HR_rest_ (bpm)	64.2 ± 7.8
HR_max_ (bpm)	182.1 ± 13.7
HR_max%_ of ageHR_max_	90.78 ± 8.33
Blood lactate @ max (mmol/L)	9.69 ± 2.35
Respiratory exchange ratio @ max	1.16 ± 0.07
RPE @ max (6–20 scale)	18.7 ± 1.0

### 2.2 Maximal graded running exercise

Maximal exercise testing was executed on a treadmill, where subjects were instructed to follow Gerkin’s graded run test (Mier and Gibson, 2004) to physical exhaustion. The criteria for maximal physiological effort were set for all subjects as blood lactate >7 mmol/L, respiratory exchange ratio (RER) > 1.15, and rating of perceived exertion (RPE) ≥17 (on a 6–20 scale), except for one female subject aged between 20 and 29 years, who had a blood lactate level of 4.4. mmol/L, a maximum RER of 1.6, an RPE of 17, and a peak ${\dot{V}}_{O_{2}}$ of 45.67 mL/kg/min, whose data were included in the analysis.

This session began with the subjects wearing the monitoring equipment. Then they were asked to sit down at rest for 7 min, at the end of which they were asked to walk on a treadmill at 0% incline at a self-selected speed (SSS) for 3 min. At the end of the SSS walking, the subjects were asked to rest for 3 min, and then they were asked to start with the maximal test until exhaustion. When exhaustion was reached, the subjects stopped or were stopped, and they were asked to sit down resting for 1 h. Blood lactate was drawn from the earlobe during the resting period before the first walking activity, immediately after stopping with the maximal test, and 1 h after the maximal test was concluded. High-resolution (20 grams) body weight was measured at the very beginning of the session two times, once when subjects were wearing sports clothes without shoes and the second time after the subjects wore the monitoring equipment and with shoes so that this second weight could be compared with the weight at the end of the exercise. The third weighing took place after drawing the blood lactate at the end of the exercise and drying the subjects thoroughly with towels. Maximal voluntary contractions (MVCs) were originally recorded before and immediately after the end of the exercise for a secondary research purpose. However, because of some technical difficulties, only a subset of subjects had usable data, and the results of the analysis were regarded as not worth reporting.

### 2.3 Submaximal constant speed running exercise

There were at least 48 h between visits. The intensity for the submaximal running exercise was determined using the V-slope method (Beaver et al., 1986) by two researchers using the maximal exercise test data. This intensity corresponded to 73% ± 10% of ${\dot{V}}_{O_{2}Max}$ . The session began with the subjects wearing the monitoring equipment and resting sitting down for 7 min. They were then asked to walk on a treadmill for 3 min at the SSS chosen during the maximal exercise session. After an additional 3 min of rest sitting down, the subjects were asked to run on the treadmill at a constant submaximal intensity. They were asked to reach a target HR by adjusting the running speed; the target HR corresponded to the HR at 80% of the anaerobic threshold intensity calculated, as mentioned above (Beaver et al., 1986). After 2–3 min of exercise intensity stabilization, the running speed was fixed for the remaining 30-min run. At the end of 30 min, the subjects were asked to rest sitting down for 3 min and then walk for 3 min at the SSS chosen during the first session and used for the pre-submaximal exercise treadmill walking activity of the current session. After another 3-min pause of resting while sitting down, the subjects were asked to walk once more for 3 min at the SSS plus 1 km/h. Finally, the subjects were asked to sit down and rest for 3 more minutes. Blood lactate was drawn from the earlobe at three time points: during the resting period before the first walking activity, immediately after the 30-min submaximal run, and at the end of the very last 3 min of recovery sitting down that followed walking at an SSS plus 1 km/h. This last walk was introduced to appreciate the effect of workload increase on the performance of the algorithm. High-resolution body weight was measured just after the subjects wore the measuring equipment at the very beginning of this session and at the end of the 30-min run after the blood lactate was drawn and the subjects were thoroughly dried with towels. As mentioned in the previous paragraph, during this session, the MVC was recorded three times in this case, i.e., before, after the exercise, and after the SSS + 1 km/h walking. Yet, because of a lack of quality data, it was decided not to report the results here.

### 2.4 Monitoring equipment

Each subject was equipped with a wearable metabolic system (K5, COSMED), by which ${\dot{V}}_{O_{2}}$ , ${\dot{V}}_{CO_{2}}$ , and respiratory volumes were monitored. Moreover, a chest-strap HR monitor (RS800CX, Polar) and a PPG and 3D accelerometry optical HR monitor (OHRM, Philips Research) were used to monitor the HR and body motion. Body temperature was inferred using a tympanic thermometer (MC510, Omron). Body weight was measured on a high-resolution scale (resolution of 20 g and capacity of 200 kg) developed and calibrated in-house (Philips Research). Finally, the whole blood lactate level was evaluated using a portable kit (Lactate Pro2, ARKRAY). Temperature, body weight (dried of sweat), and lactate levels were measured immediately before and after exercise.

### 2.5 Exertion index model

**FIGURE 2:**
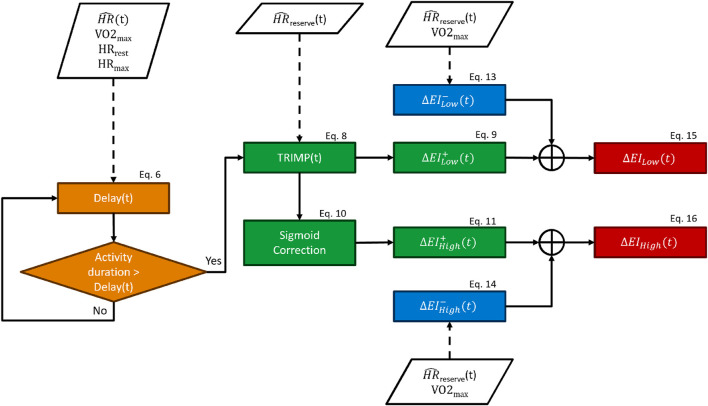
Flowchart of the heart rate-drift correction model.

The primary inputs of our model were the HR signal, the activity duration, and subjects’ characteristics like ${\dot{V}}_{O_{2}Max}$ and age. These were used to derive two exertion indexes, namely, a high-exertion index (*EI*_*High*_) and a low-exertion index (*EI*_*Low*_), which characterized each exercise intensity component. Ultimately, these exertion indexes were used to correct the measured HR for HR drift. This model consisted primarily of five computational stages: delay block, new TRIMP calculation component, sigmoid correction block, and an increase and decrease component (Figure 2). The HR correction was computed each second following the HR sampling frequency, which was set at 1 Hz in this work (Eq. 3).

The delay block forced a lag between the beginning of an exercise and the onset of the exertion indexes, where 20 is an arbitrary constant (see Eq. 6). By design, the exertion indexes began to increase only when the time spent performing the exercise was above the “delay” threshold. The *delay* function is described by Eq. 6, and it was computed for each subject and each timestamp. It can be observed in Eq. 6 that the *delay* function is dependent on the fitness level, so fitter subjects would require more time for the onset of the exertion indexes. The *delay* ranged from zero to infinity depending upon the proximity of a given HR to the HR range boundaries: the closer to resting values, the longer time required for the onset of the exertion index and *vice versa*. The boundaries were represented by ${\hat{HR}}_{\mathrm{AboveRest}}$ Eq. 4 and ${\hat{HR}}_{\mathrm{BelowMax}}$ Eq. 5. *HR*_Max_ was simply estimated using the accessible 220 - age or 230 - age in the case of subjects with physical activity rating above 5, as described by Jackson et al. (1990). *HR*_*Rest*_ was defined as the 10^th^ percentile of the HR during the trial. This block was designed to guarantee that the increase in the exertion indexes coincided with the beginning of the HR drift.

$$\hat{HR}\left(t\right)=HR\left(t\right)-\Delta HR_{\mathrm{corr}}\left(t-1\right),$$ (3)

$${\hat{HR}}_{\mathrm{AboveRest}}\left(t\right)=\left\{\begin{array}{cc} \hat{HR}\left(t\right)-HR_{\mathrm{Rest}},\; & If\;\hat{HR}\left(t\right)>HR_{\mathrm{Rest}}\\ 0,\; & Otherwise \end{array}\right.,$$ (4)

$${\hat{HR}}_{\mathrm{BelowMax}}\left(t\right)=\left\{\begin{array}{cc} HR_{\mathrm{Max}}-{\hat{HR}}_{\mathrm{Corrected}}\left(t\right),\; & If\;{\hat{HR}}_{\mathrm{Corrected}}\left(t\right)<HR_{\mathrm{Max}}\\ 0,\; & Otherwise \end{array}\right.,$$ (5)

$$delay\left(t\right)=20\cdot {\dot{V}}_{O_{2}Max}\cdot \frac{{\hat{HR}}_{\mathrm{BelowMax}}\left(t\right)}{{\hat{HR}}_{\mathrm{AboveRest}}\left(t\right)}.$$ (6)

The new TRIMP block consisted of updating the traditional TRIMP formula Eq. 2 using the corrected HR, which was used for recalculating *HR*_*Reserve*_, providing ${\hat{HR}}_{\mathrm{Reserve}}$ (Eq. 7). The constant *k* is the same as used by Desgorces et al. (2007), and *b* is the same as used by Morton et al. (1990) (Eq. 8).

The sigmoid function *Sigm* promoted a substantial accumulation of the high-intensity-related exertion index only when the TRIMP exceeded 80% of the maximum TRIMP reachable by a subject (*TRIMP*_*max*_, i.e., TRIMP calculated with the HR equal to *HR*_*max*_). The *Sigm* function was introduced to mimic the exponential increase in lactate accumulation once the exercise intensity crossed the anaerobic threshold.

We designed this model so that the increase block provided the increase in exertion indexes ( $\Delta EI_{\mathrm{Low}}^{+}$ Eq. 9 and $\Delta EI_{\mathrm{High}}^{+}$ Eq. 11) in relation to the exercise intensity, where $\Delta EI_{\mathrm{Low}}^{+}$ was directly proportional to the “new” TRIMP, while $\Delta EI_{\mathrm{High}}^{+}$ was proportional to the “new” TRIMP and was mediated by the sigmoid function described in Eq. 10.

$${\hat{HR}}_{\mathrm{Reserve}}\left(t\right)=\frac{{\hat{HR}}_{\mathrm{AboveRest}}\left(t\right)}{HR_{\mathrm{Max}}-HR_{\mathrm{Rest}}},$$ (7)

$$TRIMP\left(t\right)={\hat{HR}}_{\mathrm{Reserve}}\left(t\right)\cdot \;k\cdot \;\exp\left(b\cdot {\hat{HR}}_{\mathrm{Reserve}}\left(t\right)\right),$$ (8)

$$\Delta EI_{\mathrm{Low}}^{+}\left(t\right)=TRIMP\left(t\right),$$ (9)

$$Sigm\left(t\right)={\left[1+\exp\left(\left(0.8\cdot \;TRIMP_{\mathrm{Max}}-TRIMP\left(t\right)\right)\right.\right]}^{-1},$$ (10)

$$\Delta EI_{\mathrm{High}}^{+}\left(t\right)=Sigm\left(t\right)\cdot \;TRIMP\left(t\right).$$ (11)

The decrease block returned the decrease factors ( $\Delta EI_{\mathrm{Low}}^{-}$ , $\Delta EI_{\mathrm{High}}^{-}$ ) to be multiplied by the exertion indexes accumulated. The factors are intensity-specific and defined by Eqs 13, 14, where the *TRIMP*_*Min*_ was the average between the *TRIMP*(*t*) and the TRIMP obtained from a *HR*_*corr*_(*t*) above rest of 10 bpm. This guaranteed, through *TRIMP*_*Decr*_(*t*) Eq. 12, avoiding an excessive decrease in $\Delta EI_{\mathrm{Low}}^{-}$ and an insufficient $\Delta EI_{\mathrm{High}}^{-}$ . The time constants *τ*_*Low*_ and *τ*_*High*_ were 864,000 and 86,400 s, respectively (corresponding to 10 days and 1 day). They were chosen in order to capture the different dynamics of the two exertion indexes.

$$TRIMP_{\mathrm{Decr}}\left(t\right)=\left\{\begin{array}{cc} TRIMP\left(t\right),\; & If\;TRIMP\left(t\right)>TRIMP_{\mathrm{Min}}\left(t\right)\\ TRIMP_{\mathrm{Min}}\left(t\right),\; & Otherwise \end{array}\right.,$$ (12)

$$\Delta EI_{\mathrm{Low}}^{-}\left(t\right)=\exp\left(\frac{-{\dot{V}}_{O_{2}Max}}{\tau_{\mathrm{Low}}\cdot \;TRIMP_{\mathrm{Decr}}\left(t\right)}\right),$$ (13)

$$\Delta EI_{\mathrm{High}}^{-}\left(t\right)=\exp\left(\frac{-{\dot{V}}_{O_{2}Max}\cdot \;TRIMP_{\mathrm{Decr}}\left(t\right)}{\tau_{\mathrm{High}}}\right).$$ (14)

The *delay*, $\Delta EI_{\mathrm{Low}}^{+}$ , $\Delta EI_{\mathrm{High}}^{+}$ and $\Delta EI_{\mathrm{Low}}^{-}$ , $\Delta EI_{\mathrm{High}}^{-}$ were used in accumulated *EI*_*Low*_ and *EI*_*High*_ Eqs 15, 16 in order to obtain the current *EI*_*Low*_ and *EI*_*High*_ values:

$$EI_{\mathrm{Low}}\left(t\right)=\left\{\begin{array}{cc} \Delta EI_{\mathrm{Low}}^{-}\left(t\right)\cdot \;EI_{\mathrm{Low}}\left(t-1\right)+\Delta EI_{\mathrm{Low}}^{+}\left(t\right),\; & If\;activity\;duration>delay\left(t\right)\\ \Delta EI_{\mathrm{Low}}^{-}\left(t\right)\cdot \;EI_{\mathrm{Low}}\left(t-1\right),\; & Otherwise. \end{array}\right.$$ (15)

$$EI_{\mathrm{High}}\left(t\right)=\left\{\begin{array}{cc} \Delta EI_{\mathrm{High}}^{-}\left(t\right)\cdot \;EI_{\mathrm{High}}\left(t-1\right)+\Delta EI_{\mathrm{High}}^{+}\left(t\right),\; & If\;activity\;duration>delay\left(t\right)\\ \Delta EI_{\mathrm{High}}^{-}\left(t\right)\cdot \;EI_{\mathrm{High}}\left(t-1\right),\; & Otherwise. \end{array}\right.$$ (16)

Finally, the exertion indexes were used to remove the HR drift component from the HR according to the following equations:

$$EI_{\mathrm{Correction}}\left(t\right)=min\left(30,EI_{\mathrm{Low}}\left(t\right)+EI_{\mathrm{High}}\left(t\right)\right),$$ (17)

$$\Delta HR_{\mathrm{Correction}}\left(t\right)=\left\{\begin{array}{cc} \left(1-{\hat{HR}}_{\mathrm{Reserve}}\left(t\right)\right)\cdot \;EI_{\mathrm{Correction}}\left(t\right),\; & If\;{\hat{HR}}_{\mathrm{Reserve}}\left(t\right)<1\\ 0,\; & Otherwise, \end{array}\right.$$ (18)

$$\hat{HR}\left(t+1\right)=HR\left(t+1\right)-\Delta HR_{\mathrm{Correction}}\left(t\right).$$ (19)

The *EI*_*Correction*_ was bound to a maximum of 30 bpm in order to avoid overcorrection of the HR drift in Eq. 17. The HR drift correction (see Eqs 18, 19) was mediated by ${\hat{HR}}_{\mathrm{Reserve}}$ since the HR drift is inversely proportional to the cardiac load during the activity (Ekelund, 1967).

### 2.6 Statistical analysis

Difference analysis between uncorrected and HR drift-corrected parameters (e.g., AUCs) was performed by one-tail paired t-tests, setting the significance level at 0.05. Pearson’s linear regressions were used to assess the correlation between the biomarkers and exertion indexes. MATLAB software (MathWorks) was used for both data processing and statistical analysis.

## 3 Results

The aim of this study was to model the high (above anaerobic threshold)- and the low (below anaerobic threshold)-intensity components of the HR and ${\dot{V}}_{O_{2}}$ hysteresis. Our research hypothesis was that once the HR “drift” is properly modeled for these exercises, it can be eliminated from the HR-based ${\dot{V}}_{O_{2}}$ estimation, which otherwise would lead to a clear overestimation.

### 3.1 Heart rate–oxygen consumption hysteresis

In this investigation, we defined hysteresis as the drift of the HR in relation to ${\dot{V}}_{O_{2}}$ during and when recovering from exercise. In order to investigate this phenomenon, we normalized the HR and ${\dot{V}}_{O_{2}}$ accounting for their individual ranges.

#### 3.1.1 Maximal exercise hysteresis correction

**FIGURE 3:**
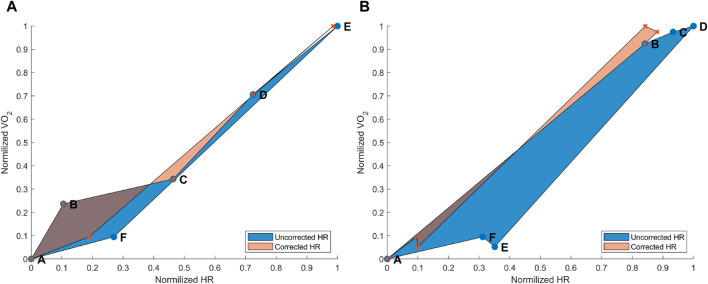
**(A)** Hysteresis derived from the maximal aerobic exercise (MAX) for a subject representative of the group. Data points: A = pre-MAX resting; B = pre-MAX SSS walking; C = beginning of MAX; D = half of MAX; E = end of MAX; and F = post-MAX resting. **(B)** Hysteresis derived from below anaerobic-threshold exercise (BTE) for a subject representative of the group. Data points: A = pre-BTE SSS walking; B = first phase of BTE (8th and 10th minute); C = middle phase of BTE (20th minute); D = end phase of BTE; E = post-BTE SSS walking; and F = post-BTE SSS + 1-km/h 2nd walking. The blue area represents the hysteresis when normalized VO_2_ is related to uncorrected normalized HR values, whereas the orange area depicts the hysteresis between the normalized VO_2_ and corrected normalized HR.

The ${\dot{V}}_{O_{2}}$ and HR values used to build the hysteresis functions for the maximal test were normalized by subtracting an offset (i.e., the mean value of 60 s taken from the pre-test resting period) from ${\dot{V}}_{O_{2}}$ and HR values and by dividing this difference by a delta obtained by subtracting the end value of the running exercise from the same offset. For the maximal exercise, we used the following points: A was the ${\dot{V}}_{O_{2}}$ and HR values corresponding to the mean value of 60 s were taken from pre-test resting, which were also used as the offset; B was the ${\dot{V}}_{O_{2}}$ and HR values corresponding to the steady-state mean value of the pre-maximal exercise SSS walking activity; C was the ${\dot{V}}_{O_{2}}$ and HR 60-s mean values were taken after 120 s from the beginning of the maximal exercise test; D was calculated as 60-s mean values of ${\dot{V}}_{O_{2}}$ and HR halfway to the end of the maximal exercise test (i.e., maximal exercise end time - maximal exercise start time/2); for E, the ${\dot{V}}_{O_{2}}$ and HR values were means of 60 s taken during the last 30 s of the maximal exercise run; and finally, F was computed by taking 60-s mean values of ${\dot{V}}_{O_{2}}$ and HR 1,200 s after the end of the exercise when the subjects were resting while sitting down (see Figure 3A, blue area). When applying the HR drift correction, the same six data points were computed as just described but with an adjusted HR, so that the hysteresis was reduced (see Figure 3A, orange area). Data from all 15 subjects were usable for this analysis. When we quantitatively analyzed the difference between the two hysteresis curves with and without HR-drift correction, we found that the AUC without correction was *AUC*_MAX_ = 0.099 ± 0.060, and the AUC after the HR-drift correction was *corrAUC*_MAX_ = 0.071 ± 0.056; the correction did significantly reduce the AUC when the HR-drift correction was applied [t(14) = 3.396, *p* = 0.002]. At a group level, the AUC decreased by 26.14% ± 27.54%, but for three subjects, it increased by 33.45, 4.80, and 13.98%, respectively.

#### 3.1.2 Submaximal exercise hysteresis correction

The ${\dot{V}}_{O_{2}}$ and HR values used to build the hysteresis functions for the submaximal exercise were normalized by subtracting an offset (i.e., mean steady-state value of SSS walking executed for the submaximal exercise; first 30 s were removed) from ${\dot{V}}_{O_{2}}$ and HR values and dividing this difference by a delta obtained by subtracting the end value of the submaximal running exercise from the same offset. For the submaximal exercise hysteresis, we used the following points: A was the ${\dot{V}}_{O_{2}}$ and HR values corresponding to the mean steady-state value of SSS walking executed for the submaximal exercise after removing the first 30 s, which was also used as the offset; B was the ${\dot{V}}_{O_{2}}$ and HR values corresponding to the mean value of 120 s starting at the 8th minute of the exercise up to the 10th minute; C was the ${\dot{V}}_{O_{2}}$ and HR 120-s mean values of 20 min after the start of the submaximal exercise; D was calculated at the end of the exercise taking the mean of 120 s and 30 s before the end of the submaximal exercise; for E, ${\dot{V}}_{O_{2}}$ and HR values were means of the SSS walking (i.e., speed chosen during the first session) after removing 30 s at the beginning and the end of the walking activity; and finally, F was computed by taking the means of the second post-submaximal running exercise walk (i.e., this was at the SSS + 1 km/h) after removing 30 s at the beginning and the end of the walking activity (see Figure 3B, blue area). When applying the HR drift correction, the same six data points were computed as described in the paragraph above but with an adjusted HR, with the aim to reduce the hysteresis (see Figure 3B, orange area). Data from 11 subjects were usable for this analysis. When we quantitatively analyzed the difference between the two hysteresis curves with and without HR-drift correction, we found that the AUC without correction was *AUC*_*BTE*_ = 0.241 ± 0.145, and the AUC after the HR-drift correction was *corrAUC*_*BTE*_ = 0.058 ± 0.056; the correction did significantly reduce the AUC when the drift correction was applied [t(10) = 6.299, *p* < 0.001]. At a group level, the AUC decreased by 77.00% ± 10.29%, and it decreased for all subjects (11 of 11).

### 3.2 Heart rate-drift correction

**FIGURE 4:**
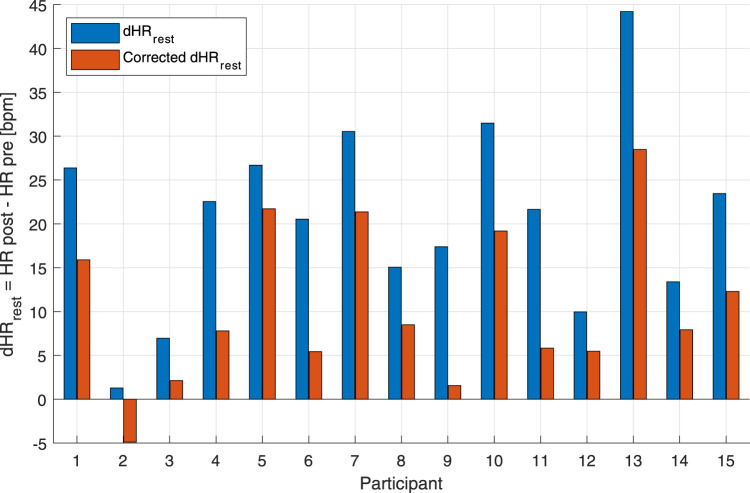
Bar graph of uncorrected and corrected HR drifts of the individual subjects for the maximal aerobic exercise (MAX) session. Blue bars represent the difference between post-MAX resting and the pre-MAX resting of the uncorrected HR. Orange bars show the difference between post-MAX resting and the pre-MAX resting of the corrected HR.

**FIGURE 5:**
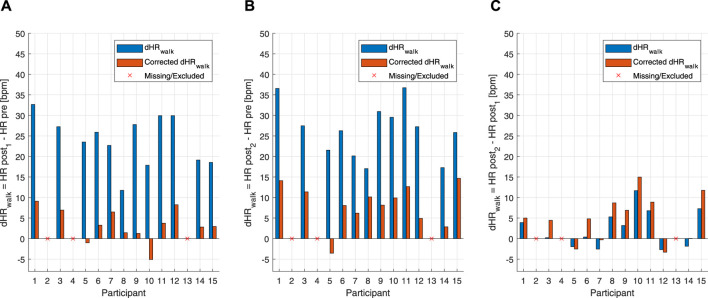
Bar graph of uncorrected and corrected HR drifts of the individual subjects for the below anaerobic-threshold exercise (BTE) session. **(A)** Blue bars represent the difference between post-BTE SSS walking (post 1) and the pre-BTE SSS walking (pre) of the uncorrected HR. Orange bars represent the difference between post-BTE SSS walking (post 1) and the pre-BTE SSS walking (pre) of the corrected HR. **(B)** Blue bars represent the difference between post-BTE SSS + 1-km/h walking (post 2) and the pre-BTE SSS walking (pre) of the uncorrected HR. Orange bars represent the difference between post-BTE SSS + 1-km/h walking (post 2) and the pre-BTE SSS walking (pre) of the corrected HR. **(C)** Blue bars represent the difference between post-BTE SSS + 1-km/h walking (post 2) and post-BTE SSS walking (post 1) of the uncorrected HR. Orange bars represent the difference between post-BTE SSS + 1-km/h walking (post 2) and the post-BTE SSS walking (post 1) of the corrected HR.

An additional way to test the performance of the combined *EI*_*low*_ and *EI*_*high*_-based HR correction model was to calculate HR deltas between pre-exercise and post-exercise states. For the MAX, uncorrected and corrected post-resting and pre-resting differences were tested using a right-tailed paired *t*-test. This showed that the HR drift for the corrected HR (10.57 ± 9.07 bpm) was significantly lower than that for the uncorrected original HR (20.77 ± 10.77 bpm) [t(14): 8.739, *p* < 0.001] (Figure 4). For the BTE deltas that analyzed the difference between HRs post- and pre-BTE SSS walks, the corrected drift was significantly lower (6.16 ± 4.03 bpm) than the uncorrected original drift (23.92 ± 3.34 bpm) [t(11): 14.391, *p* < 0.001] (Figure 5A). The same was found for the difference between HRs post-BTE SSS + 1 km/h and pre-BTE SSS walks, where the uncorrected drift (26.39 ± 8.29 bpm) was reduced significantly (6.58 ± 5.18 bpm) [t(11): 11.040, *p* < 0.001] (Figure 5B). Finally, and probably more interestingly, the difference between the uncorrected (2.47 ± 4.64 bpm) and corrected HR (4.95 ± 5.69 bpm) of the two post-BTE walks, namely, post-BTE SSS + 1 km/h and post-BTE SSS walks, resulted in significantly greater delta for the corrected values [t(11): −4.794, *p* < 0.001] (Figure 5C). This was to be expected because the difference between the uncorrected HR of the two post-BTE walks accounts for two phenomena: i) a workload increase of 1 km/h (e.g., + 5 bpm) and ii) a slow decay in the HR drift (e.g., −3 bpm). Meanwhile, theoretically, in the case of the corrected HR, only the workload increase was accounted for.

#### 3.2.1 High- and low-intensity components for HR-drift correction

**FIGURE 6:**
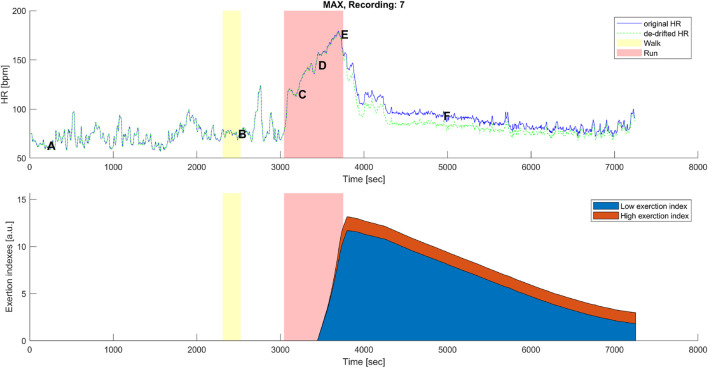
Top panel: original (blue) and corrected (green) HR time series before, during, and after the maximal aerobic exercise (MAX) of a representative subject. Data points A, B, C, D, E, and F are as described in Figures 1A, 3A. Bottom panel: cumulative time series of the low-exertion index (blue) and the high-exertion index (red) as a result of the activities performed during the MAX session. The yellow banner indicates the pre-MAX walking activity at a self-selected speed. The red banner indicates the MAX activity.

**FIGURE 7:**
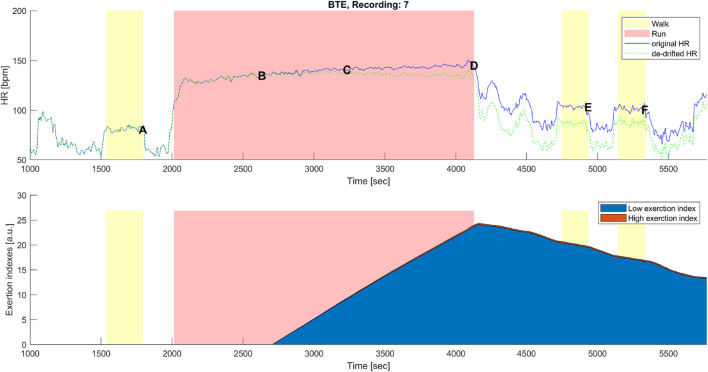
Top panel: original (blue) and corrected (green) HR time series before, during, and after the below anaerobic-threshold exercise (BTE) of a representative subject. Data points A, B, C, D, E, and F are as described in Figures 1B, 3B. Bottom panel: cumulative time series of the low-exertion index (blue) and of the high-exertion index (red) as a result of the activities permed during the BTE session. The yellow banner indicates all walking activities, pre- and first post- at a self-selected speed, while the third yellow bar indicates walking at the self-selected speed plus 1 km/h. The red banner indicates the BTE activity.

A distinctive feature of the correction model presented in this work was the coexistence of two components, *EI*_*Low*_ and *EI*_*High*_. The low exertion index was designed to detect below-threshold exercises and those high above the threshold. The qualitative analysis given in Figures 6, 7 shows that HR-drift correction (green dashed line in the top panels) does not onset as soon as the exercises start, as intended by the *dealy* function. In the bottom panels of Figures 6, 7, *EI*_*High*_ is clearly greater than *EI*_*Low*_ for the MAX activity, and the opposite is true for the BTE. However, the magnitude of *EI*_*High*_ and *EI*_*Low*_ is greater for BTE because the exercise duration is far greater than *dealy*, accounting for greater HR drifts.

### 3.3 Correlations with lactate levels and water loss

**FIGURE 8:**
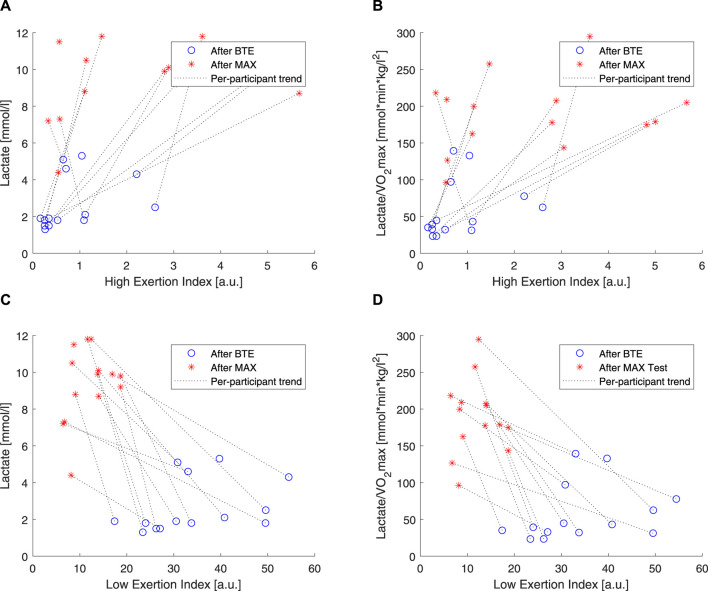
Correlations of lactate and high exertion index (EI) and low EI. Blue open circles refer to below anaerobic-threshold exercise (BTE), red asterisks refer to maximal aerobic exercise (MAX), dotted lines represent linear trendlines.

To understand how the two components (i.e., *EI*_*Low*_ and *EI*_*High*_) of the exercise exertion model actually related with the low- and high-exercise intensities, linear correlations were performed (Figure 8). It is clear that *EI*_*High*_ is approximately 10 times smaller than *EI*_*Low*_. Moreover, as expected, the lactate levels, chosen here as a marker of anaerobic exertion, were higher after MAX activity than BTE, and *EI*_*High*_ clustered around lower values for BTE running. Meanwhile, *EI*_*Low*_ showed a very clear divide, displaying greater values for the BTE activity. In order to check whether cardiorespiratory fitness would affect these indexes, lactate levels were normalized by ${\dot{V}}_{O_{2}Max}$ , but this showed no change in those correlations.

**FIGURE 9:**
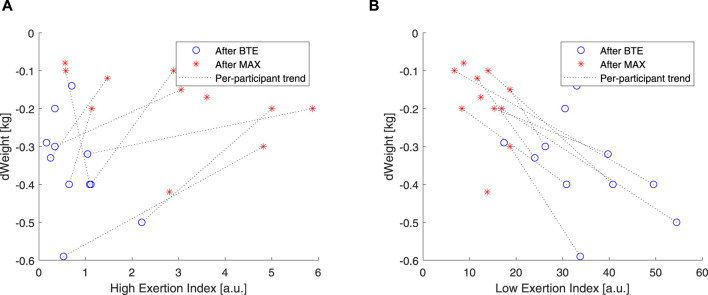
Correlations of weight and high EI and low EI. Blue open circles refer to below anaerobic-threshold exercise (BTE), red asterisks refer to maximal aerobic exercise (MAX), dotted lines represent linear trendlines.

As introduced in this article, it is well documented that CVD is affected by dehydration. A simple way to estimate exercise dehydration is weight loss when no solid or fluid intake and excretion (e.g., feces and urine) are ensured. Under these conditions, weight loss is due to water loss + CO_2_ expiration. According to our ${\dot{V}}_{CO_{2}}$ measurements, we expect weight loss due to CO_2_ expiration to be approximately 70 g for the MAX running and 225 g for the BTE. Measurement errors could not be excluded as the weighing scale resolution was 20 g, and the procedure of drying off sweat may have caused external errors. Therefore, weight loss values below 70 g for MAX and 225 g for BTE were not considered valid. We observed that weight loss for MAX running seemed less pronounced than that for the BTE running sessions (Figure 9). *EI*_*Low*_ showed a much stronger relation to dehydration (i.e., weight loss) than *EI*_*High*_.

## 4 Discussion

We developed an HR-based model that can significantly correct for HR drift during and after maximal and submaximal exercises by using cardiovascular fitness information, exercise activity intensity, and duration.

As expected, MAX running did not show an evident HR drift during the incremental exercise test itself (see Figure 1A, points B–E). However, an evident HR elevation was present during the recovery phase, which can be appreciated by observing the horizontal distance between points A and F in Figure 1A. In most cases, ${\dot{V}}_{O_{2}}$ elevation (e.g., slow component) was observable during the recovery phase. This can be appreciated from the hysteresis Figure 1A when point F is above 0 on the *y*-axis (i.e., ${\dot{V}}_{O_{2}}$ ). The hysteresis figure of BTE prolonged running activities confirmed the occurrence of an HR drift during and after the exercise. This could be seen by observing the horizontal shift toward the right from point B to point D, which represented a constant workload (see Figure 1B). As for the MAX test and the BTE case, a light ${\dot{V}}_{O_{2}}$ drift was observable at point E, often not back at the level of point A on the *y*-axis. However, on the *x*-axis, a clearer HR drift could be observed during recovery, as shown by the distance between points E and A on this axis (see Figure 1B). Our proof-of-concept model showed that the hysteresis could be reduced by an instantaneous HR correction. Quantitatively, we showed that our model reduced the AUC of the hysteresis for the MAX session by 26% and for the BTE session by 77%. In the MAX activity, HR drift was present only during recovery, whereas in the BTE activity, it was present for both exercise and recovery phases, and this cumulative effect could explain the greater AUC correction our model performed for BTE. Our model corrected the HR drift in all subjects for both MAX and BTE activities, as shown in Figures 4, 5. Interestingly, when the two post-BTE walking activities were compared, one at SSS and the other at SSS + 1 km/h, we observed that our model corrected only for the increase in workload (see Figure 5C).

Our model was designed so that it started correcting for HR drift only when the exercise intensity was high enough and when the duration was prolonged enough. For instance, short and very intense exercise would not impact the dehydration component of the drift. On the other hand, long but very mild exercise (e.g., walking) would require far more time to produce a dehydrating effect. Since *EI*_*High*_ and *EI*_*Low*_ indexes are HR-based, the model should theoretically work when mild exercise is performed under exceptional conditions (e.g., very hot and dry) that significantly affect the HR. In this study, the EI correction accounted for the high-intensity exercises via the *EI*_*High*_ component, which, on the one hand, could rapidly increase but, on the other hand, did not have the time to reach high magnitudes (see Figure 5), whereas the EI correction during lower intensities, *EI*_*Low*_ component, such as for BTE running, had a smaller slope of increase, but it had the time to reach greater (2-fold) magnitudes (see Figure 7).

In order to evaluate how these two components, high and low, related to the exercise intensity and duration, we used two rather “simple” markers. We used blood lactate levels as markers of anaerobic exercise exertion (Faude et al., 2009), and we correlated these to our *EI*_*High*_ and *EI*_*Low*_. Our results showed that *EI*_*High*_ was particularly unresponsive to BTE running, but it did show some spread in response to the MAX sessions (see Figures 8A, B). *EI*_*Low*_ was clearly, by design, able to increase 10 times more than *EI*_*High*_, and higher levels were shown in response to BTE sessions (Figures 8C, D). Moreover, cardiovascular fitness did not seem to influence these relationships (Figure 9A did not differ from Figure 9B). Exercise-induced dehydration was estimated by weight loss. Although we experienced some difficulties in obtaining reliable weight loss measurements in all subjects, by setting some boundaries (i.e., Δ*weight* >70 g for MAX and Δ*weight* >125 g for BTE), we attempted limiting the measurement errors and drawing wrong conclusions. We observed that weight loss for MAX running seemed less pronounced than for the BTE running sessions. *EI*_*Low*_ showed a much stronger relation to dehydration (i.e., weight loss) than *EI*_*High*_. This was in accordance with our expectations. The lactate and the weight loss evidence seemed to confirm that *EI*_*Low*_ better reflected the below-anaerobic threshold exercise, whereas *EI*_*High*_ reflected the above-anaerobic threshold.

Souissi et al. (2021) recently reviewed the causes of CV drift, concluding that it has a multifactorial nature. For prolonged exercise, hyperthermia, dehydration, hypovolemia, and consequent SV decrease (thus, HR increase) are known causes. However, there are more reasons for CV drift than HR drift alone, for example, decrease in left ventricular compliance (Souissi et al., 2021). In our study, HR drift was clearly present during the submaximal exercise and its recovery and during recovery from maximal exercise. HR elevation above resting levels is well documented; for instance, Facioli et al. (2021) showed that even cardiovascularly fit subjects needed more than 36 min to recover from submaximal exercise at 90%–95% of *HR*_max_. We designed our model so that it would mimic similar kinetics.

The main implication of our proof-of-concept work is that whenever in the future ${\dot{V}}_{O_{2}}$ or ${\dot{V}}_{O_{2}Max}$ or energy expenditure are estimated using the HR, HR drift must be at least acknowledged and best accounted for. Several limitations exist in this work. First, being a proof-of-concept model, our model is based on certain arbitrary decisions, such as the constants *k* and *b* taken from the literature (Morton et al., 1990; Desgorces et al., 2007) or the arbitrary constant of 20 in the delay (Eq. 6). Second, we used only two types of exercise and one modality, while in the future, the model should be tested under diverse conditions, such as above-threshold but submaximal exercise, high-intensity interval exercise, and in modalities like cycling. Additionally, we limited the monitoring period to 1 h after the end of the maximal exercise test, but it would be interesting to investigate how this model would perform after many hours and even days. Water loss was only indirectly estimated by measuring body weight using a high-resolution scale before and after exercise. However, wiping sweat off the participants was not an error-free process. We tried to mitigate these errors by setting a minimum for CO_2_ loss. Yet, these results should be considered with caution. Although we collected free-living data (not shown and disclosed in this work), a general limitation in those types of investigation is assessing ${\dot{V}}_{O_{2}}$ under free-living conditions. All these considerations should inspire future research.

## 5 Conclusion

In this study, differences in maximal and submaximal exercise-induced ${\dot{V}}_{O_{2}}$ and HR kinetics were graphically described by a hysteresis relation. The proof-of-concept model encompassing both low- and high-intensity exercise exertion showed a significant reduction in the hysteresis area during exercise and consequent recovery. This model allowed HR drift instantaneous correction, which could be exploited in the future for achieving improvement in HR-based ${\dot{V}}_{O_{2}}$ estimates.

## Data Availability

The datasets presented in this article are not readily available because of concerns about participant privacy and confidentiality. Requests to access the datasets should be directed to FS, francesco.sartor@philips.com.
